# Commentary: Impact of evidence-based nursing intervention based on Watson's theory on rehabilitation compliance in elderly patients after intertrochanteric femur fracture surgery

**DOI:** 10.3389/fmed.2026.1905365

**Published:** 2026-08-14

**Authors:** Lihua Luo

**Affiliations:** Department of Orthopedics, Sichuan Modern Hospital, Chengdu, Sichuan, China

**Keywords:** enhanced recovery after surgery, evidence-based nursing, intertrochanteric femur fracture, psychological mediators, rehabilitation compliance

Zhang et al. report a prospective randomized controlled trial in 80 elderly patients with intertrochanteric femur fractures, in which an evidence-based nursing (EBN) intervention built on Watson's theory of human caring was associated with higher rehabilitation compliance, fewer complications, faster recovery, and better quality of life and hip function than routine care (1). The study is thoughtfully designed around a coherent theoretical framework and tackles a genuinely difficult problem—poor rehabilitation adherence in elderly fracture patients. I read it with interest and, in a constructive spirit, offer several observations intended to help in interpreting the present findings and in shaping future work.

To orient readers who have not consulted the original article, the study was a single-center, prospective randomized controlled trial in which 80 elderly patients (aged 60–80 years) with intertrochanteric femur fractures, all treated with proximal femoral anti-rotation intramedullary nailing, were allocated by a random-number-table method to routine perioperative nursing (control, *n* = 40) or to a Watson's-theory-based EBN bundle in addition to routine care (observation, *n* = 40). The bundle, delivered by an interdisciplinary team that included orthopedic physicians, a clinical psychologist and specialist nurses, comprised a healing environment with personalized care plans, daily empathic psychological nursing, individualized pain management, and multidimensional social support with post-discharge follow-up, over a 3-month intervention period. Rehabilitation compliance—the study's primary outcome, assessed

with a self-developed questionnaire—was the principal endpoint; secondary outcomes included pain on a visual analog scale (VAS) at 3 days, 7 days and 1 month after surgery, weight-bearing walking time, fracture healing time and length of stay, quality of life (WHOQOL-BREF), nursing satisfaction, the Harris hip score (HHS), and complications, with quality of life and hip function measured at baseline and at 3 months.

First, I noticed an apparent inconsistency in the reporting of the pain outcomes that may warrant clarification. The abstract and the Results text state that visual analog scale (VAS) scores were lower in the observation group at 3 days, 7 days and 1 month, with *P* < 0.01. Table 2 of the original article, however, lists *P*-values of 0.137, 0.020, and 0.043 at these three time points, respectively. On the tabulated figures, the 3-day difference is not statistically significant, and the 7-day and 1-month differences, although significant, do not reach the *P* < 0.01 threshold cited in the text. I suspect a transcription error and would gently encourage the authors to reconcile the text, abstract and table, or to issue a correction if appropriate, since the statement that the intervention lowers early post-operative pain rests on this comparison. It may also be worth noting that the absolute between-group differences in VAS are modest (for example, 0.68 points at 1 month), which invites a cautious reading of their clinical, as distinct from statistical, significance.

Second, a note on the analysis of the outcomes measured before and after the intervention. Quality of life (WHOQOL-BREF) and the Harris hip score were each assessed at baseline and at 3 months, yet the groups were compared on their post-intervention values using independent-samples *t*-tests. Baseline scores were almost identical between groups, which is reassuring; even so, analysis of covariance (adjusting the follow-up score for its baseline) or comparison of change scores is generally preferable, as it makes use of the paired structure of the data and protects against any residual imbalance. In addition, the numerous outcomes were each tested separately without adjustment for multiplicity; designating primary and secondary endpoints, or applying a correction, would give readers added confidence that the uniformly significant results are robust. Relatedly, although the authors report a power analysis, it was performed *post-hoc* on an assumed effect size rather than as an a priori calculation anchored to a clearly pre-specified primary outcome; given the modest sample (n = 80) and the number of endpoints examined, an a priori calculation tied to a single primary outcome would place the inferences on firmer ground. These are refinements of analysis and presentation rather than doubts about the overall direction of the findings.

Third, it may be worth reflecting on the robustness of the largest effects. The most striking outcomes—rehabilitation compliance (90.0% vs. 37.5%) and satisfaction (92.5% vs. 42.5%)—were captured with locally developed questionnaires, and, as the authors transparently acknowledge, the intervention providers could not be blinded, although outcome assessors and analysts were blinded and patients submitted their responses anonymously. Self-reported adherence and satisfaction are, by their nature, the measures most sensitive to expectation effects, and they are also where the between-group differences here are largest. I raise this not to question the result but to suggest that corroborating it with a more objective index of adherence—for example, documented attendance at supervised rehabilitation sessions or simple activity monitoring—would make an already compelling finding still harder to dispute.

Fourth, and at the heart of the study, the intervention is fundamentally psychological: it works through empathic communication, a helping–trusting relationship, and the easing of fear, anxiety and kinesiophobia so as to build self-efficacy. The authors interpret their findings in just these terms and, in their limitations, call for work exploring “physiological–psychological mediating mechanisms.” I would add that the very constructs proposed as the mediators—anxiety, depression, kinesiophobia and self-efficacy—were not themselves measured. Where such affective outcomes have been quantified, orthopedic enhanced-recovery and EBN programmes demonstrably relieve perioperative anxiety and depression (2) and reduce post-operative delirium (3), consistent with the long-standing recognition that the surgical stress response these programmes attenuate is psychological as well as metabolic and inflammatory (4, 5). As a direction for future research, measuring these mediators directly with validated instruments, examining them as pre-specified mediators, and reporting the psychological domain of the WHOQOL-BREF separately from the total score, would allow future studies to show, rather than infer, that the benefit travels along the pathway the theory predicts.

Finally, because the protocol is intensive—an interdisciplinary team including a clinical psychologist, structured staff training, and daily empathic sessions—these approaches lie beyond the scope of the present 80-patient trial and are offered as directions for future research; a factorial design paired with these mediator measurements would help identify which caring components carry the effect and support the development of a scalable form suited to busy, resource-limited wards. None of these observations diminishes the contribution of Zhang et al., who have brought a well-articulated caring theory to bear on a hard clinical problem with encouraging results; offered in a collegial spirit, they are meant only to strengthen the case for psychologically informed nursing in elderly orthopedic care. I thank the authors for a stimulating study.
